# The prospective effect of fucoidan on splenic dysfunction caused by oxaliplatin in male rats through endoplasmic stress dynamics

**DOI:** 10.1038/s41598-022-25441-6

**Published:** 2022-12-22

**Authors:** Eman H. Basha, Amira M. ElShamy, Hoda A. Ibrahim, Mohamed A. Safa, Nehal A. E. Heabah, Radwa Awad, Radwa Ismail, Rabab M. Amer, Ola M. Salem, Heba Faheem, Yasmeen M. El-Harty

**Affiliations:** 1 Department of Medical Physiology, Faculty of Medicine, Tanta University, Tanta, Egypt; 2 Department of Medical Biochemistry, Faculty of Medicine, Tanta University, Tanta, Egypt; 3 Department of Internal Medicine, Faculty of Medicine, Tanta University, Tanta, Egypt; 4 Department of Pathology, Faculty of Medicine, Tanta University, Tanta, Egypt; 5 Department of Clinical Oncology, Faculty of Medicine, Tanta University, Tanta, Egypt; 6 Department of Anatomy, Faculty of Medicine, Tanta University, Tanta, Egypt; 7 Department of Medical Pharmacology, Faculty of Medicine, Tanta University, Tanta, Egypt

**Keywords:** Biochemistry, Physiology, Anatomy, Medical research

## Abstract

Fucoidans (FUCs) are highly sulfated polysaccharides demonstrating multiple actions in different systems. Oxaliplatin (OXA) is a platinum-containing chemotherapeutic agent with several side effects that restrict its usage. The current study aimed to determine the potential effect of FUC in male rats with splenic dysfunction induced by OXA. Eighty adult male rats aged (8–9 weeks) weighing (190–230 g) were divided into four groups: (Group I: the control group): Rats were administrated normal saline; (Group II: controls treated by FUC): Rats were treated with FUC; (Group III: Splenic dysfunction group): Rats were treated with 8 mg/kg OXA. (IV: Splenic dysfunction treated by FUC): Rats were treated by OXA as Group III, then fucoidan was given. At the end of the experiment, blood was collected to determine red blood cells and white blood cells. Splenic tissues were divided into one part for biochemical assays, oxidative stress markers as MDA and catalase, inflammatory markers (TNF-alpha, IL6), and apoptotic markers (caspase 3) and gene expression of *Nrf2*, *Mapk1* gene expression, and endoplasmic stress parameters and the other part was used for immunohistochemical and histopathological analysis. Compared to the OXA-induced splenic dysfunction group, FUC significantly decreased high levels of MDA, TNF- alpha, IL6, caspase-3, *Mapk1*, endoplasmic stress induced by OXA, and increased the level of catalase and *Nrf2*. Fucoidan has corrected the histopathological and immunohistochemical changes compared to the OXA-induced splenic dysfunction group. In conclusion, our findings suggest that fucoidan has a significant role in the treatment of splenic dysfunction induced by OXA.

## Introduction

Fucoidans (FUCs) are highly sulfated polysaccharides isolated from the cell walls of different species of brown seaweeds, such as *Saccharina japonica*, and certain animal species like *Sea cucumber*^1^.

FUCs have many bioactions indicated by several previous studies, including hypoglycemic, antioxidant, anti-inflammatory, anticoagulant, and antiviral effects, and represent a functional food capable of performing additional systemic effects^2^.

Due to the potential variations in its chemical structure, the biological effects of FUC vary from species to species. FUC is not constant and varies from one type to the other based on species source of isolation, as its components as uronic acid, d-xylose, d-mannose, and d-galactose vary by species, indicating a global risk of FUC in the industry for several pathological conditions^3,4^.

The previously studied therapeutic effects of FUC, its non-toxic nature, biocompatibility altogether with its curative effects in arthritis, liver diseases, brain diseases, Crohn's disease, and ulcerative colitis, in addition to its role in suppressing fibrosis while maintaining cellular integrity in many pathological conditions, strongly elicit research questions regarding the significant role of FUC in treatment and prevention of these disorders as well as other diseases^5^.

The spleen can play an essential role in body functions such as filtration of blood, immunity, phagocytosis, iron metabolism, removal of infectious substances, and disturbed RBCs (Red blood cells). In addition, the spleen represents the largest lymphoid tissue. Consequently, splenic dysfunction impairs a variety of biological body functions^6^.

OXA, a platinum-containing chemotherapeutic agent generally used as first- or second-line treatment for colorectal cancer^7^, is an alkylating agent that induces cytotoxicity via intracellular hydrolysis. The platinum compound binds to DNA, forming cross-links that inhibit DNA replication and transcription, resulting in cell death which stimulates apoptosis^8^.

Although OXA is highly effective against many cancers, its side effects are the main causes of dose limitation, presenting an obstacle to effective treatment of cancer especially gastrointestinal side effects, peripheral neuropathy, and hematological toxicity^9^.

Interestingly, the pathophysiological mechanisms that underlie OXA-induced spleen dysfunction are not fully understood^10^ but previous studies confirmed that OXA causes oxidative stress (OS), inflammation, apoptosis, and fibrosis through different mechanisms^11^.

It is necessary to decrease the side effects of OXA and enhance its anti-cancer efficacy, which can occur by combining OXA with natural products^12^.

As a result, we anticipate that FUC could have a role in splenic dysfunction by improving the inflammatory, apoptotic, oxidative, and endoplasmic reticulum stress caused by OXA.

## Material and methods

### Experimental animals

The study was carried out on 80 adult male rats of the local strain 6 month weighing (190–230 g). The rats were housed in standard well-ventilated animal cages at room temperature, with free access to water and food throughout the entire period of work. The maximum number of rats per cage was assigned to three to avoid cage overcrowding or decreased cleanliness. Rats were monitored five times a week for signs of cage aggression. All procedures were done according to the ethical committee of Tanta University (Approval Code Number: 34448/2/21) and in accordance with ARRIVE guidelines.

### Drugs and chemicals

FUC was supplied by Sigma-Aldrich Co, (Louis, MO).

OXA was supplied by Sigma-Aldrich Co, (Louis, MO).

All these drugs were dissolved in saline.

### Animal groups

After 1 week of acclimatization, the rats were divided into four groups (20 rats each):

Group I: Control group: The rats were treated with intraperitoneal injection of 0.5 mL of normal saline weekly for 8 weeks.

Group II: Control treated by FUC: The rats were treated with FUC 100 mg/kg daily which was given by intraperitoneal injection for 8 weeks^13^.

Group III: Splenic dysfunction: The rats were treated with 8 mg/kg OXA 0.5 mL which was given by intraperitoneal injection weekly for 8 weeks.

Group IV: Splenic dysfunction treated by FUC: The rats were treated with 8 mg/kg OXA 0.5 mL which was given as the third group plus FUC 100 mg/kg daily was given by intraperitoneal injection for eight weeks that was given concurrently at the same time with OXA that FUC^11^.

### Blood sample and biochemical assessment of blood cells

At the end of the experimental period, male rats were anesthetized by intraperitoneal injection of pentobarbital (50 mg/kg)^14^, sacrificed by cervical dislocation, and blood samples were obtained by decapitation of all animals, then transferred to tubes containing Ethylenediamine tetraacetic acid (EDTA) as anticoagulant, weight blood cells (WBCs) and RBCs counts were determined.

### Preparation of splenic tissues

The splenic tissues of animals, from each studied group, were randomly divided into three divisions. One division seven samples was assigned for biochemical sample analyses after proper homogenization. Another division 6 samples was assigned for real time gene expression analysis. The third division seven samples was assigned for histopathological and immunohistochemical changes analyses.

### Biochemical analysis of inflammatory status, OS and apoptosis

The assigned tissue samples were homogenized in cold phosphate buffer (pH 7.4), then centrifugated at 3000 rpm for 10 min. The resulting supernatants were separated in clean storage plastic test tubes and stored at – 80 °C to be used for immunoassay determination of tumor necrosis factor-α (TNF-α) (Cat# No MBS355371) (sensitivity < 1 pg/mL) and interleukin-6 (IL-6) (Cat# No MBS726707) (sensitivity 1.0 pg/mL)as markers of inflammation by ELISA kits.

Also, colorimetric assay was carried out for determination of MDA level as OS marker, in accordance with the methodology described by Ref.^15^, Catalase as an antioxidant marker (Cat# No: ab83464), Nitric oxide (NO) level (Cat# No: E-BC-K035-M) (sensitivity 0.16 μmol/L), NO concentration was indirectly estimated by nitrate/nitrite detection, Caspase-3 activity as a marker of apoptosis (Cat# No: ab39401) and (#Cat 500–0006, Bio-Rad Protein Assay) was used to measure splenic tissue homogenates total proteins.

Quantitative estimation of Endoplasmic reticulum stress parameters (*GRP78*, *CHOP* and *DPP4* findings), Mitogen activated protein kinase (*Mapk1*), Nuclear factor erythroid2 related factor (*Nrf2*) relative genes expression by real time PCR.

### Real-time PCR

Total RNA was extracted from frozen splenic tissue after processing using Qiagen RNeasy Total RNA isolation kit (Qiagen, Hiden, Germany) according to the protocol provided by the manufacturer. By a NanoDrop spectrophotometer (NanoDrop Technologies, Inc. Wilmington, USA), The total RNA concentration and purity were measured at the OD260 and OD260/280 ratios, respectively. RNA has an A260/A280 ratio of 1.9–2.2 and the RNA was then preserved at-80° C. This was followed by synthesis of the first strand using Super-Script III First-Strand Synthesis System for real-time PCR kit (Life Technologies, Carlsbad, California, USA) according to manufacturer's instructions. PCR reactions were performed using Power SYBR Green PCR Master Mix (Life Technologies, Carlsbad, California, USA) following the manufacturer's instructions. MAPK, Nrf2, GRP78, CHOP and DPP4 mRNA transcripts were quantified relative to the housekeeping gene GAPDH gene, which was used as an internal control. Sequence specific primers were designed (Table 1). The thermal cycling conditions were as follows: Initial denaturation at 95 °C for 10 min was followed by 40 cycles with denaturation at 95 °C for 15 s, annealing at 60 °C for 30 s and extension at 72 °C for 30 s. At the end of the last cycle, the temperature was increased from 60 to 95 °C for melting curve analysis. Relative gene expression was automatically calculated using the comparative threshold (Ct) method for the values of the target and the reference genes using the 2^−ΔΔCT^ formula.

**Table 1:** Sequence specific primers.

Gene	Gene no.	Primers	Amplicon size
*Mapk1*	001164043.1	Forward: 5′-AGGGCGATGTGACGTTT-3′ Reverse: 5′-CTGGCAGGGTGAAGTTGG-3′	1123
*Nrf2*	010902.5	Forward: 5′-CAGTGCTCCTATGCGTGAA-3′ Reverse:: 5′-GCGGCTTGAATGTTTGTC-3′)	1776
*GRP78*	14866.1	Forward: 5′-GGA GGA TGT GGG CAC GGT GGTC-3′ Reverse:: 5′-GTC ATT CCA AGT GCG TCC GAT GAGG-3′	1064
*CHOP*	001109986.1	Forward: 5′-AGGAGAGAGAAACCGGTCCAA-3′ Reverse:: 5′--3′	2983
*DPP4*	012789.1	Forward: 5′-CCAACTCCAGAGGACAACCT-3′ Reverse:: 5′-TCTTCGTCCGTGTACCACAT-3′	197
*GAPDH*	031144.3	Forward: 5′-CACGATGGAGGGGCCGGACTCATC-3′ Reverse: 5′-TAAAGACCTCTATGCCAACACAGT-3	241

### Histopathological evaluation of splenic tissues

Splenic samples were fixed in 10% formalin and embedded in paraffin. Five µm sections of formalin fixed paraffin embedded (FFPE) blocks were stained with hematoxylin and eosin (H&E) for routine histopathological evaluation. Slides were examined for histopathological changes such as architectural abnormality, inflammatory response, vascular congestion, and apoptosis.

### Immunohistochemical analysis

Tissue sections were deparaffinized in xylene and dehydrated with graded concentrations of alcohol. Antigen retrieval was done by immersion in citrate buffer (pH 6.0) for 10 min at 95 °C. Then the tissue samples were naturally incubated with 0.3% H_2_O_2_ for 15 min to block the endogenous peroxidase activity.

After washing in phosphate-buffered saline (PBS), incubation with anti-bcl-2 antibodies (C-2: sc-7382, Santa Cruz Biotechnology, INC, USA, dilution 1:100) was done, for 2 h at room temperature. Slides were then washed and incubated biotinylated secondary antibodies for 20 min. After washing, sections were incubated with diaminobenzidine substrate-chromogen solution (DAB) for 30 s. Finally, all sections were counterstained with Mayer's hematoxylin. Negative controls were performed by replacing the primary antibody with PBS.

### Assessment of bcl-2 immunohistochemical staining

Cytoplasmic staining was interpreted as positive for bcl-2. Bcl-2 scoring was as follows, considering ten high power fields (400×): score 0 (no staining), score + 1 (weak positive, staining of < 10% of the cells), score + 2 (moderate, staining of 10–75% of the cells), and score + 3 (strong positive, staining of > 75% of the cells). Scores of 0 and + 1 were considered negative, and scores of + 2 and + 3 were considered positive for bcl-2 expression^16^.

### Morphometric study

Five non-overlapping random fields (magnification: 400×; area: 0.071 mm^2^) of splenic tissue sections were chosen, photographed, and submitted for morphometric study. Quantification of optical color density and the mean area percentage of bcl2 immunohistochemical positive cells in DAB-stained sections was performed using Image software (Media Cybernetics).

### Statistical analysis

The obtained results were represented using the mean ± standard deviation. One-way ANOVA and Tukey's post hoc test were used to analyze and evaluate the significance. Statistical significance was considered at p-values < 0.05. SPSS software (Version 23.0, IMB, NY) was used for statistical analyses.

### Ethics approval

Animals used in these experiments were treated in accordance with the procedures approved by the ethical committee of Tanta University (Approval Code Number: 34448/2/21). No human blood sample was included.

### Resarch involving human participants and/or animals

This work did not include any human blood samples but only animal samples.

## Results

### Effect of FUC on oxidant/antioxidant biomarkers, apoptosis and inflammatory biomarkers

The splenic catalase level was decreased significantly in OXA-treated group in comparison to other studied groups (*P* < 0.05) while MDA and NO levels showed the reverse. FUC co-treatment significantly increased catalase level and significantly decreased MDA and NO levels (*P* < 0.05), Inflammatory biomarkers TNF-α and IL-6 levels showed a significant increase in OXA treated group when compared to other studied groups (P < 0.05). On the other hand, FUC co-treatment significantly decreased all the aforementioned inflammatory biomarkers (P < 0.05),splenic tissue caspase 3 level showed a significant increase in OXA treated group when compared to other studied groups (P < 0.05). On the other hand, FUC co-treatment significantly decreased caspase 3 level in splenic tissue (P < 0.05) (Table 2).

**Table 2:** Effect of FUC on oxidant/antioxidant biomarkers, apoptosis and inflammatory biomarkers among the studied groups.

Groups	Group I	Group II	Group III	Group IV	F value
TNF-α (pg/mg protein)	54.34 ± 0.73^ cd^	56.05 ± 1.45 cd	83.5 ± 2.74 ^abd^	70.51 ± 2.28^abc^	341.58
IL-6 (pg/mg protein)	25.58 ± 1.22^c,d^	24.93 ± 0.45^c,d^	51.93 ± 1.12 ^a,b,d^	35.95 ± 0.82 ^a,b,c^	1232.51
MDA (mmol/g tissue)	10.07 ± 0.12^c,d^	9.76 ± 0.72^c,d^	15.95 ± 0.65^a,b,d^	12.15 ± 0.57^a,b,c^	176.77
NO (µM/g tissue)	42.32 ± 1.15^c,d^	42.82 ± 1.37^c,d^	65.36 ± 1.32^a,b,d^	53.8 ± 1.36^a,b,c^	489.69
Catalase (u/g tissue)	31.35 ± 1.54^c,d^	31.33 ± 3.66^c,d^	14.44 ± 1.76^a,b,d^	24.79 ± 1.64^a,b,c^	82.72
Caspase3 (μM/mg tissue)	3.87 ± 0.64^c,d^	4.47 ± 0.3^c,d^	24.76 ± 1.0^a,b,d^	16.39 ± 0.6^a,b,c^	1483.63

Data are represented as mean ± SD. Statistical analysis was carried out using one-way ANOVA with Tukey's post hoc test, SPSS computer program. ^a–d^Significant difference between groups at *p < 0.05. ^a^Significance from group I; ^b^significance from group II; ^c^significance from group III; ^d^significance from group IV. Degree of freedom is (3) between groups and (24) within groups (total = 27). *TNF-α* Tumor necrosis factor-alpha, *IL-6* Interleukin-6, *MDA* MDA, *NO* Nitric oxide.

### Effect of FUC on some hematological parameters (RBCs and WBCs counts)

RBCs and WBCs counts showed a significant decrease (anemia and leucopenia, respectively) in OXA treated group when compared to other studied groups (P < 0.05). On the other hand, FUC co-treatment significantly increased RBCs and WBCs counts (P < 0.05) (Table 3).

**Table 3:** Effect of FUC on hematological parameters (RBCs and WBCs) counts among the studied groups.

Groups	Group I	Group II	Group III	Group IV	F value
RBCs (10^6^ cells/mm^3^)	6.8 ± 0.4^c,d^	6.7 ± 0.4^c,d^	3.5 ± 0.2 ^a,b,d^	4.46 ± 0.2^a,b,c^	144.09
WBCs (10^3^ cells/mm^3^)	11.1 ± 0.4^c,d^	10.9 ± 0.4^c,d^	6.8 ± 0.4 ^a,b,d^	8.3 ± 0.2 ^a,b,c^	166.88

Data are represented as mean ± SD. Statistical analysis was carried out using one-way ANOVA with Tukey's post hoc test, SPSS computer program. ^a–d^Significant difference between groups at *p < 0.05. ^a^Significance from group I; ^b^significance from group II; ^c^significance from group III; ^d^significance from group IV. Degree of freedom is (3) between groups and (24) within groups (total = 27). *RBCs* Red blood cells, *WBCs* White blood cells.

### Effect of FUC on *mRNA* gene expression

**Figure 1:**
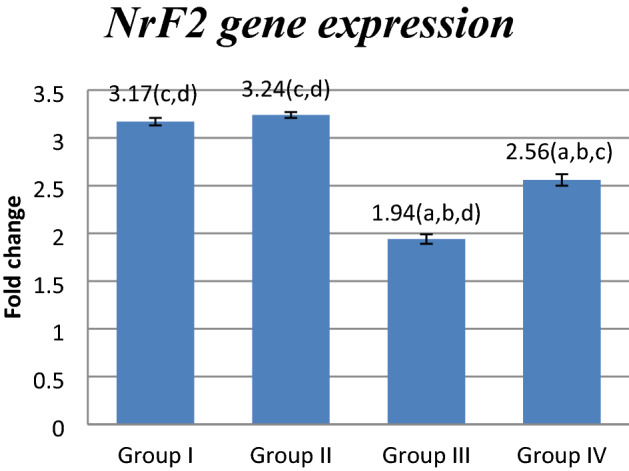
Effect of FUC treatment on *NrF2 mRNA* gene expression. values are represented as mean ± SD. Data are represented as mean ± SD. Statistical analysis was carried out using one-way ANOVA with Tukey's post hoc test, SPSS computer program. ^a–d^Significant difference between groups at *p < 0.05. ^a^Significance from group I; ^b^significance from group II; ^c^significance from group III; ^d^significance from group IV. Degree of freedom is (3) between groups and (24) within groups (total = 27).

**Figure 2:**
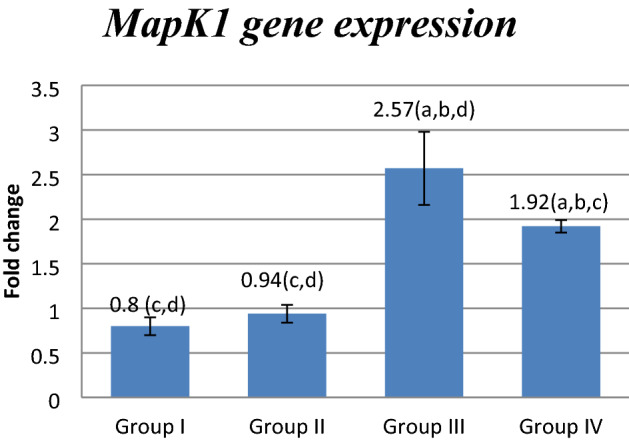
Effect of FUC treatment on *Mapk1 mRNA* gene expression. values are represented as mean ± SD. Data are represented as mean ± SD. Statistical analysis was carried out using one-way ANOVA with Tukey's post hoc test, SPSS computer program. ^a–d^Significant difference between groups at *p < 0.05. ^a^Significance from group I; ^b^significance from group II; ^c^significance from group III; ^d^significance from group IV. Degree of freedom is (3) between groups and (24) within groups (total = 27).

**Figure 3:**
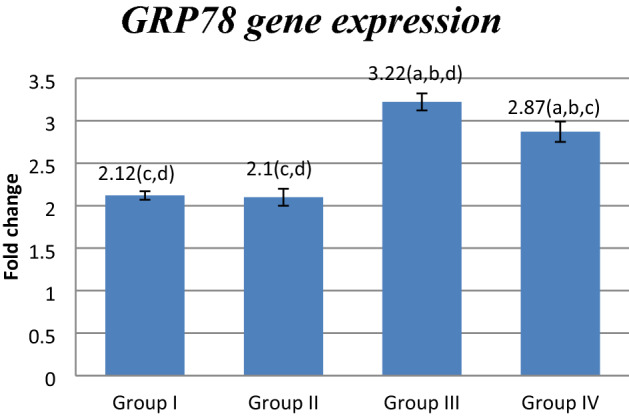
Effect of FUC treatment on *GRP78 mRNA* gene expression. values are represented as mean ± SD. Data are represented as mean ± SD. Statistical analysis was carried out using one-way ANOVA with Tukey's post hoc test, SPSS computer program. ^a–d^Significant difference between groups at *p < 0.05. ^a^Significance from group I; ^b^significance from group II; ^c^significance from group III; ^d^significance from group IV. Degree of freedom is (3) between groups and (24) within groups (total = 27).

**Figure 4:**
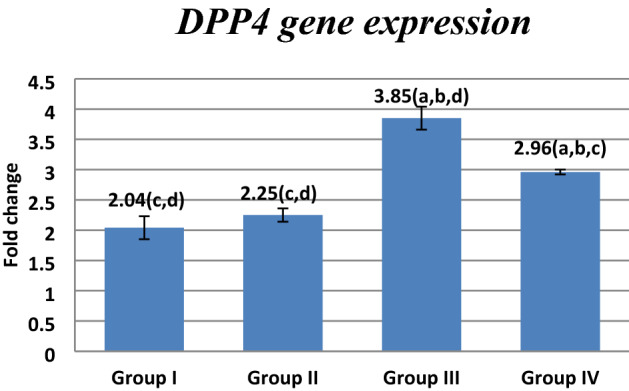
Effect of FUC treatment on *DPP4 mRNA* gene expression. values are represented as mean ± SD. Data are represented as mean ± SD. Statistical analysis was carried out using one-way ANOVA with Tukey's post hoc test, SPSS computer program. ^a–d^Significant difference between groups at *p < 0.05. ^a^Significance from group I; ^b^significance from group II; ^c^significance from group III; ^d^significance from group IV. Degree of freedom is (3) between groups and (24) within groups (total = 27).

**Figure 5:**
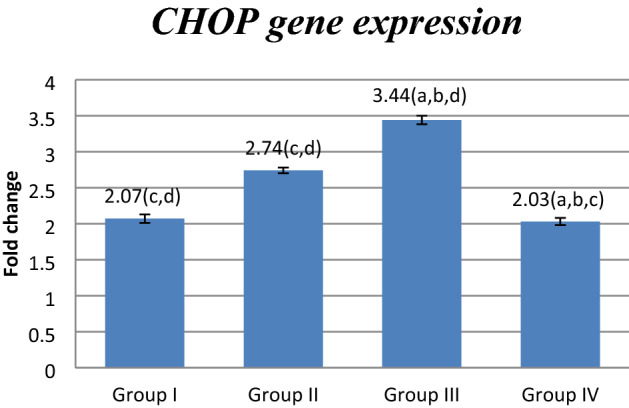
Effect of FUC treatment on *CHOP mRNA* gene expression. values are represented as mean ± SD. Data are represented as mean ± SD. Statistical analysis was carried out using one-way ANOVA with Tukey's post hoc test, SPSS computer program. ^a–d^Significant difference between groups at *p < 0.05. ^a^Significance from group I; ^b^significance from group II; ^c^significance from group III; ^d^significance from group IV. Degree of freedom is (3) between groups and (24) within groups (total = 27).

MAPK, GRP78,CHOP and DPP4 mRNA gene expression showed a significant increase in OXA treated group when compared to other studied groups (P < 0.05) while NrF2 mRNA gene expression showed the reverse.FUC co-treatment significantly decreased MAPK, GRP78,CHOP and DPP4 m RNA gene expression and significantly increased NrF2 mRNA gene expression(*P* < 0.05) (Figs. 1, 2, 3, 4, 5).

### Histopathological results

**Figure 6:**
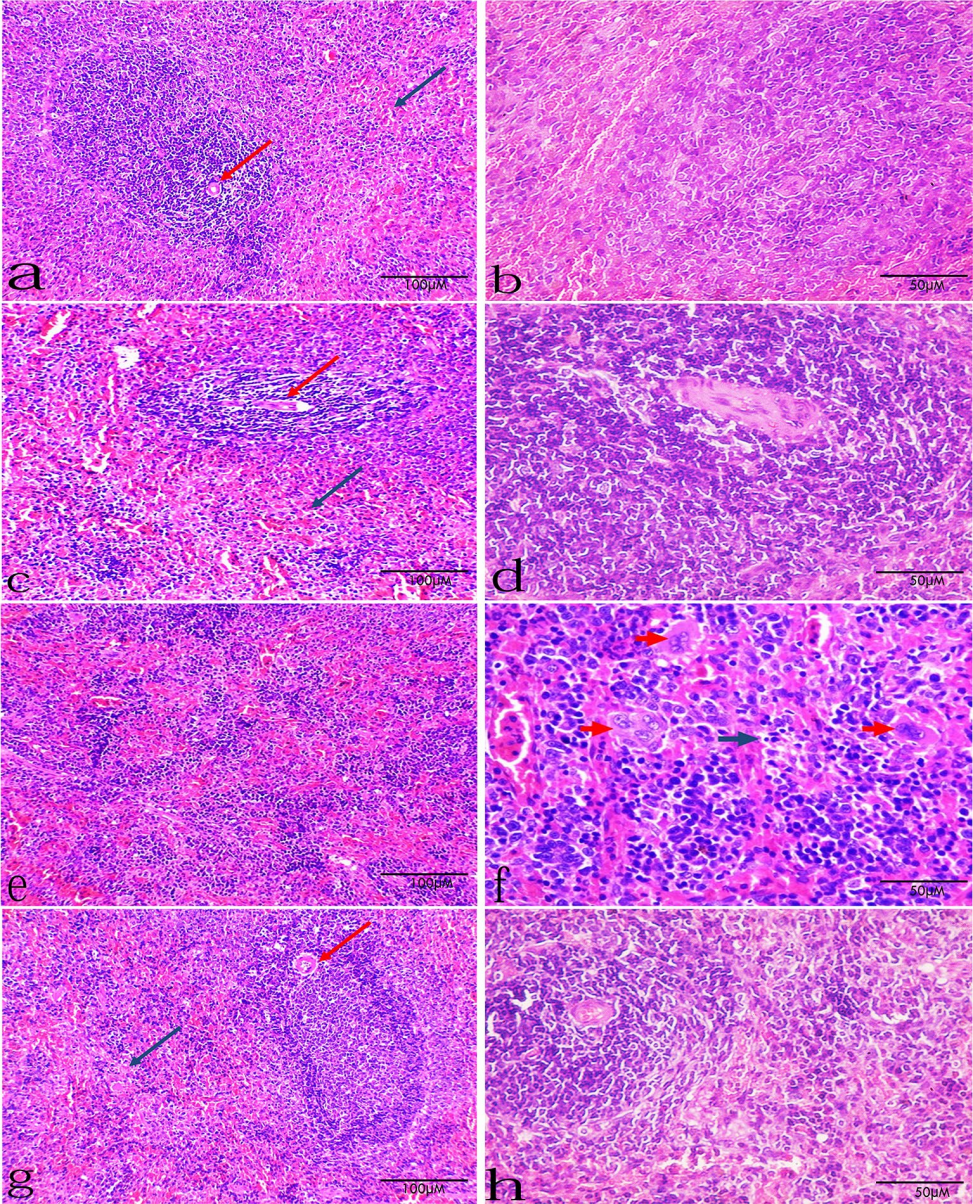
Group I (**a**,**b**): Splenic tissue in a control rat showing normal architecture with normal red pulp (blue arrow) and white pulp with central arteriole (red arrow). Group II (**c**,**d**): Splenic tissue in a control rat treated by FUC showing normal architecture with normal red pulp (blue arrow) and white pulp with central arteriole (red arrow). Group III (**e**,**f**): Splenic tissue in a splenic dysfunction rat showing disturbed architecture in the form of irregular white pulp and congested red pulp. Its higher magnification showing red pulp with extramedullary hematopoiesis with many megakaryocytes (red arrows) and apoptotic bodies (blue arrow). Group IV (**g**,**h**): Splenic tissue in a splenic dysfunction treated by FUC rat showing preserved architecture with clearly defined red pulp (blue arrow) and white pulp with central arteriole (red arrow). The left panel is with low magnification: ×200, scale bar 100 μm. and the right panel is with high magnification: ×400, scale bar 50 μm.

Splenic tissue in the control group showed normal architecture with normal red pulp and white pulp with central arteriole. Also, the control treated by FUC group showed normal architecture of splenic tissue. Splenic tissue in the group III (Splenic dysfunction) showed disturbed architecture in the form of irregular white pulp and congested red pulp. Its higher magnification showed red pulp with extramedullary hematopoiesis with many megakaryocytes and apoptotic bodies. Splenic tissue in the group IV (Splenic dysfunction treated by FUC) showed preserved architecture with clearly defined red pulp and white pulp with central arteriole (Fig. 6).

### Immunohistochemical and morphometric results

**Figure 7:**
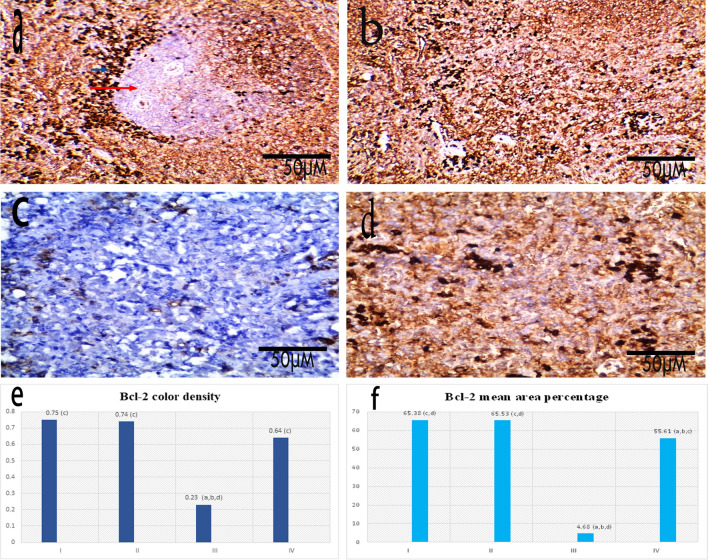
(**a**) Group I: Bcl-2 expression in spleen of a control rat showing positive expression of the red pulp as well as the mantle zone of the white pulp (blue arrow), while the germinal center of the white pulp showed negative bcl-2 expression (red arrow) [× 200]. (**b**) Group II: Bcl-2 expression in spleen of a control rat treated by FUC showing positive expression of the red pulp [× 200]. (**c**) Group III: Bcl-2 expression in spleen of a splenic dysfunction rat showing negative expression of the red pulp [× 400]. (**d**) Group IV: Bcl-2 expression in spleen of a splenic dysfunction treated by FUC rat showing positive expression of the red pulp [× 400], scale bar 50 μm. (**e**,**f**) Graphic representation of the morphometric results of Bcl-2 color density and the mean area percentage. Statistical analysis was carried out using one-way ANOVA with Tukey's post hoc test, SPSS computer program. (**a**–**d**) Significant difference between groups at *p < 0.05. (**a**) Significance from group I; (**b**) significance from group II; (**c**) significance from group III; (**d**) significance from group IV.

Bcl-2 expression in splenic tissue in the control group showed positive expression of the red pulp as well as the mantle zone of the white pulp while the germinal center of the white pulp showed negative bcl-2 expression. Also, Bcl-2 expression in the control treated by FUC group showed positive expression of the red pulp. Bcl-2 expression in the group III (Splenic dysfunction) showed negative expression of the red pulp. Bcl-2 expression in the group IV (Splenic dysfunction treated by FUC) showed positive expression of the red pulp (Fig. 7a–d). There was a significant decrease in Bcl-2 color density and the mean area percentage in group III (Splenic dysfunction) (0.23 ± 0.036, 4.68 ± 2.34) respectively compared to the control group (0.75 ± 0.17, 65.38 ± 3.5 respectively (p < 0.05). It increased significantly to normal levels in group IV (0.64 ± 0.089, 55.61 ± 4.15) respectively compared to group III (p < 0.05) (Fig. 7e,f).

## Discussion

The current study demonstrated that 8-week FUC treatment significantly treats splenic dysfunction caused by OXA in male rats as shown by our biochemical, histopathological, and immune-histochemical results.

Our results showed that rats of OXA-induced splenic dysfunction group showed significantly increased OS which is evidenced by the significant increase of MDA and NO level and significant decrease of catalase.

Excessive levels of reactive oxygen species (ROS) damages the cells directly by eliciting lipid peroxidation, protein degradation with subsequent DNA damage. Notably, whenever the ROS production exceeds the ability of the cell's natural antioxidant defenses to sequester, OS develops^17^.

The OXA-induced OS is related to several factors, including the fact that OXA causes mitochondrial dysfunction by damaging mitochondrial DNA and interrupting RNA expression of Cytochrome B, resulting in disruption of mitochondrial function necessitating antioxidant therapy^18^.

Furthermore, mitochondrial damage activates nitric oxide synthase (NOS), and NO's direct toxicity is significantly increased through the reaction with superoxide and eventually yielding into peroxynitrite, that in turn changes protein's tyrosines into nitrotyrosines, resulting in severe OS^9^.

Our results also showed that OXA-treated splenic tissue exhibit significantly decreased expression NRF2 also indicating OS. NRF2, a central transcription factor, plays a crucial role in producing antioxidant and detoxifying enzymes. The physiological binding of Nrf2 to Ketch-like ECH-associated protein (Keap1) is disrupted by OS with subsequent upregulation of the transcription of antioxidant response element (ARE)-controlled genes. This in turn upregulates the expression of a number of antioxidant and phase II drug metabolizing enzymes, such as HO-1 and NQO1, which inhibit OS^19^.

Another factor contributing to the OXA-induced splenic dysfunction was found to be the increased splenic cell apoptosis as indicated by the significant increase of caspase3 and significant decrease of BCL-2, demonstrated in the current study, together with the previously reported upregulated expression of apoptosis-related proteins, such as Bax. The OXA-induced activation of both inflammatory and apoptotic pathways was also evidenced in the current study by the significantly increased splenic *Mapk1* protein expression.Bcl-2, a member of the Bcl-2 protein superfamily, is found on the outer mitochondrial membrane preventing the mitochondrial liberation of cytochrome C and other pro-apoptotic molecules into the cytosol^20^.

On the other hand, the extracellular signal-regulated kinase, c-Jun N-terminal kinase (JNK), and p38 *Mapk1* pathways are all members of the *Mapk1 *family. The *Mapk1 *signaling pathway induces p38 phosphorylation, activates transcription factors eventually speeding up the apoptotic process indicating the ROS-induced upregulation of the JNK and p38 MAPK pathways^12^.

Previous studies explained that the OXA-induced apoptosis is attributed to the increased ROS production, Nox1-associated with JNK/p38-MAPK activation and survivin degradation by p38 activation^21^.

This causes many proapoptotic events, including p53 activation, Bax translocation, cytochrome c release, and activation of caspase-3 and 9. Also, OXA upregulates the calcium N channel which increases the intracellular calcium accumulation aiding the apoptotic mechanisms^18^.

Moreover, After OXA therapy, the antiapoptotic protein Bcl-2 level is significantly decreased, Bcl-2 is a member of the Bcl-2 protein superfamily, which includes antiapoptotic proteins. Bcl-2 is found on the outer mitochondrial membrane and prevents the release of pro-apoptotic molecules from the mitochondria to the cytosol such as cytochrome C^15^.

Another suggested mechanism for OXA-induced apoptosis is that it is capable of inhibiting tumor-associated NADH oxidase (tNOX) thus, decreasing the cell's NAD+/NADH ratio and inhibiting the SIRT1 deacetylase activity via the tNOX-induced modulation of the NAD+-SIRT1 axis that results in apoptosis^22^.

The current study reported OXA-induced inflammatory changes in splenic tissue as indicated by the significant increase of TNF-α and IL-6. The developed inflammatory process contributed to the splenic tissue damage and apoptosis as aforementioned. In agreement with our results^17^, reported the markedly increased splenic TNF-α, IFN-γ and IL-17 in OXA-treated mice with subsequent potent cytotoxic immune response, apoptosis and splenic injury.

The complex interdependent relationship between inflammatory, OS and apoptosis triggered by OXA treatment can be furtherly explained as follows. TNF-α activates caspase-3, which causes necrosis and triggers the apoptotic pathway. OS stimulates many inflammatory pathways, such as nuclear factor (*NF-κB*) and NLR family pyrin domain containing 3 (*nlrp3*), with subsequent upregulated expression of inflammatory cytokines. Moreover, the aforementioned ROS-induced triggering of the JNK and caspase pathways results also in apoptosis induced by TNF-α^17^.

Furthermore, our results revealed an OXA-associated ER stress, which is evidenced by the significantly increased expression of protein levels of *GRP78*, *CHOP,* and *DPP4* in the splenic tissue. These three proteins, also known as the unfolded protein response (UPR) or ER stress response, are the mediators of the signaling cascade responsible for increasing the protein folding capacity in response to ER stress as a method of restoration of the cell's homeostasis^23^. Moreover, ROS excess is also linked to ER stress as reported by Ref.^24^, which implies that OXA treatment with apoptosis is a common sequence to ER stress and activation of *the CHOP* pathway^23^.

Interestingly, besides splenic dysfunction, OXA treatment induced a significant decrease in RBCs and WBCs, as demonstrated in the current study. Also, in vitro toxicity of OXA was reported in the form of the emergence of polychromatophilic reticulocytes or malformed RBCs, such as spherocytes which were attributed to OXA's ability to bind with hemoglobin and cytokine secreted during cellular stress indicating DNA damage^10^.

The effect of OXA on splenic tissue is evident in animals and humans, and the histopathological results of the current study agree with other previous reports. Histopathological examination of the OXA group showed disturbed architecture and apoptotic bodies, so splenic dysfunction. These changes reflect the chemical changes detected in splenic tissue homogenate.

The results of the current study showed that FUC could alleviate OS, indicated by the significant decrease of MDA and significant increase of catalase. The significant upregulation of splenic NRF2 protein expression also evidences FUC's ameliorative effect on OS.

According to previous studies, FUC is a powerful antioxidant and radical scavenger. The relation between the content of sulfate and radical capacity in scavenging superoxides was positive, and the ratio of sulfate to FUC activity was an effective indicator^5^.

Those reports were supported by the work done by Ref.^25^, which denoted that the FUC active sulfate group enhances the antioxidant ability of FUC that, in turn, can stop Fenton's reaction and ROS generation by chelating transition metal ions necessary for a free radical chain reaction in addition to metal complexes formation.

In a further analysis, the neutralization of NO free radical at 1 mg/ml FUC was fully shown in S. polycystum, which indicates that FUC has a high NO scavenging ability^5^.

These mechanisms are in a parallel line with^26^, who added that FUC inhibited LPO, decreased NO level, and increased the antioxidants near to normal values.

There is no contradiction between these results and the report by Ref.^1^ that FUCs help scavenges ROS such as hydroxyl, peroxyl, and superoxide radicals and enhance SOD, catalase, and glucose6 phosphate dehydrogenase (G6PD).

In the present study, FUC induced significantly upregulated expression of Nrf2. FUC-induced increased GSK-3β phosphorylation provides an explanation for these results, especially since glycogen synthase kinase-3β (GSK-3β) is the upstream molecular of the Nrf2 signaling pathway^27^.

FUC also showed a potent anti-inflammatory activity indicated by the significant decrease in TNF-α and IL-6 in splenic tissue. Furtherly explaining, FUC was reported to inhibit leukocytic recruitment, block L-selectin, and hinder the cell–cell interactions, which is evident in our results as well as the results by Ref.^28^ in the form of absent inflammatory cell infiltration in comparison with the intoxicated rats as confirmed by results with^26^.

In accordance with its aforementioned anti-inflammatory effect, the current study demonstrated FUC-induced downregulated expression of NFκB, which is confirmed by the findings of Ref.^1^ regarding the downregulation of NFκB, protein kinase B, extracellular signal-regulated kinase, c-Jun N-terminal kinase, and p38 mitogen-activated protein kinase expression. In addition, it reduced LPS-enhanced elevation of serum levels of TNF-α, IL-1β, and IL-6 in mice, and it reclines Seth's induced PGE2 and IL-6 plasma levels caused by aspirin.

Moreover, our results also demonstrated that FUC-induced downregulated expression of splenic MAPKs. This finding can be explained by the inhibition of ROS production by FUC, which are an important trigger for *Mapk*s activation^29^.

Interestingly, our work also demonstrated that FUC is an antiapoptotic, evidenced by the decreased caspase 3 level and increased expression of Bcl-2, which was confirmed by the immunohistochemical staining. These results are in accordance with the work done by Ref.^30^, who demonstrated that FUC decreased hepatocyte apoptosis with up-regulation of p42/44 *Mapk *dependent NDRG-1/CAP43 and VMP-1.

In addition, FUC inhibited intrinsic and extrinsic apoptosis mediated by the TRADD/TRAF2 and JAK2/STAT1 pathways that are induced by TNF-α and IFN-γ. These reports represented methods to protect against splenic apoptosis and dysfunction^31^.

Furthermore, FUC treatment induced significant downregulation of expression of protein levels of *GRP78*, *CHOP,* and *DPP4* in the splenic tissue evidencing improvement of the ER stress. The decrease in ER stress can be attributed to FUC's efficient inhibition of the *MAPKs* activation, namely (p-JNK and p-P38), as evidenced in our data as well as the report by Ref.^29^.

Other data suggested that FUC protects splenic dysfunction through the activation of AKT and *Mapk*, with subsequently improved ER stress^29^.

Furthermore, the present study showed that FUC significantly increased WBCs and RBCs count, which supports the previous findings reporting that FUC is a stimulator of hematopoiesis^32^.

These results were parallel with the previous reports that include many mechanisms, including mobilizing leucocytes from bone marrow to peripheral blood, enhanced differentiation of the mobilized CD34+ cells into leucocytes, and the antibacterial and antiviral properties of FUC^33^.

Eventually, FUC treatment successfully ameliorated these pathological effects, as evidenced by showing normal architecture with normal red pulp (blue arrow) and white pulp of splenic tissue in the FUC treated group, which confirms the aforementioned improved splenic tissue parameters.

## Conclusion

We concluded that FUC could protect against splenic dysfunction caused by OXA by targeting endoplasmic stress dynamics and improving OXA-induced oxidative, inflammatory, and apoptotic stress in splenic tissue.

### Recommendations

Our data suggest that FUC can be used as a promising adjuvant therapy to ameliorate splenic dysfunction caused by OXA. However, further research is required to fully understand its safety limits, toxicity, and effects on splenic tissue.

### The limitations and strengths of this study

Our data suggest that fucoidan can be used as a promising prophylactic therapy to ameliorate splenic damage with OXA administration. However, further research is required to understand its safety limits fully and toxicity, study its effect as a treatment rather than prophylaxis of the toxic effect of OXA on the spleen and verify the mechanism of action of its preservative effect on the leucocytic and RBCs count. In addition, the mechanism of the effect of fucoidan on apoptosis needs further investigation to determine if it is directly or indirectly mediated through its effect on ER dynamics.

## Data Availability

All data that support the finding of the current study are available from the corresponding author upon reasonable request. Data sharing applies to this article as new data were analyzed in this study.
